# Are students kidding with health research ethics? The case of HIV/AIDS research in Cameroon

**DOI:** 10.1186/1472-6939-13-12

**Published:** 2012-06-11

**Authors:** Nchangwi Syntia Munung, Godfrey B Tangwa, Chi Primus Che, Laurent Vidal, Odile Ouwe-Missi-Oukem-Boyer

**Affiliations:** 1 University of Buea, Buea, Cameroon; 2 Centre International de Référence “Chantal BIYA” pour la Recherche sur la Prévention et la Prise en charge du VIH/SIDA (CIRCB), Yaoundé, BP, 3077, Cameroon; 3 University of Yaoundé I, Yaoundé, Cameroon; 4 Institut de Recherche pour le Développement, Marseille, France; 5 Cameroon Bioethics Initiative (CAMBIN), Yaoundé, Cameroon; 6 Current address: Centre de Recherche Médicale et Sanitaire (CERMES), BP 10887, Niamey, Niger

## Abstract

**Background:**

Universities in Cameroon are playing an active part in HIV/AIDS research and much of this research is carried out by students, usually for the purpose of a dissertation/thesis. Student theses/dissertations present research findings in a much more comprehensive manner and have been described as the stepping-stone of a budding scientist’s potential in becoming an independent researcher. It is therefore important to verify how students handle issues of research ethics.

**Method:**

Theses/dissertations on HIV/AIDS that described research studies involving the use of human research participants were screened to verify if research ethics approval and informed consent were obtained and documented. The contents of the consent forms were also qualitatively analyzed.

**Results:**

Of 174 theses/dissertations on HIV, ethics approval was documented in 17 (9.77%) and informed consent in 77 (47.83%). Research ethics approval was first mentioned at all in 2002 and highly reported in the year 2007. Evidence of ethics approval was found for the first time in 2005 and informed consent first observed and evidenced in 1997. Ethics approval was mostly reported by students studying for an MD (14.01%) and was not reported in any Bachelors’ degree dissertation. Informed consent was also highly reported in MD theses (64.58%) followed by undergraduate theses (31.58%). Voluntary participation and potential benefits of the study were some of the common aspects dealt with in most of the consent forms. The right to discontinue participation in the study and management of residual samples were scarcely ever mentioned.

**Conclusions:**

Overall, and given the current state of the art of research ethics around the world, student-scientists in Cameroon would seem to be merely kidding with research ethics. It is thus essential that training in health research ethics (HRE) be incorporated in the curriculum of universities in Cameroon in order that the next generation of scientists may be better equipped with thorough knowledge and practice of HRE. This, we believe, would be one way of fighting the occurrence of research scandals, which have not yet abated significantly, especially those arising from negligence or inexcusable ignorance.

## Background

HIV/AIDS is a serious public health problem in Cameroon, with the country having an HIV prevalence rate of 5.1% (UNAIDS: AIDS epidemic update. November, 2009). The fight against the pandemic in Cameroon is multi-sectorial and research institutions, particularly universities and medical schools, are playing an active role through scientific research. In these academic institutions, much of the research is carried out by students, usually for the purpose of a dissertation/thesis. The nature of such research varies and is usually in the biomedical sciences, clinical sciences, epidemiology and behavioral sciences (Munung et al., unpublished).

Compared to journal articles, student theses and dissertations enjoy greater word count allowances and present research findings in a more comprehensive manner. Student theses/dissertations have been described as a stepping-stone and reflection of a student’s potential in becoming an independent researcher [1,2]. It is therefore important to verify how students handle issues of Health Research Ethics (HRE), as documenting research ethics compliance in dissertations could be an appropriate start in improving the level and skill of reporting ethical aspects in scientific write-ups [2]. In addition to research ethics being of paramount importance in health-related research, the case of HIV research needs to be highlighted, as people living with HIV/AIDS are rightly considered a highly vulnerable population. The Council for International Organizations of Medical Sciences (CIOMS) Guidelines requires that special care be taken when recruiting vulnerable populations into a study and that measures should be put in place to protect the welfare and rights of such persons. This would be largely achieved if research proposals go through thorough and objective review by a Research Ethics Committee (REC) or Institutional Review Board (IRB) and if valid and appropriate informed consent is obtained from all research participants.

The literature on bioethics in Cameroon has focused primarily on philosophical discussions and opinions [3-9] and the few empirical studies that exist have centered on capacity building of RECs and members of RECs [10-12], while some have discussed ethics and Cameroon journals [13]. Worldwide, studies pertaining to the documentation of ethics approval and informed consent have been dominated by reviews of published journal articles [14-16] and similar studies had been described in Cameroon which reveal that authors of scientific journal articles of research carried out in Cameroon significantly failed to document research ethics approval and informed consent in their publications [17,18]. The same study also reveals that, in addition to the national ethics committee of Cameroon, most of the state universities in the country and some hospital centers actually had a REC or an IRB [17].

Previous studies on research ethics pertaining to student dissertations have been carried out in Sri Lanka [19], Sweden [2,20] and Turkey [21], and the last two had focused on nursing dissertations. These studies had showed that a large number of students failed to document research ethics approval and informed consent in their theses/dissertations [19,21]. To the best of our knowledge there exist no studies on the documentation of research ethics approval and informed consent in students’ theses/dissertations in Central Africa and Cameroon in particular. We therefore looked at student theses/dissertations on HIV for information and evidence of research ethics approval and informed consent as well as the content of consent forms that were used to enroll research participants. This study describes for the very first time the status and trend of research ethics in students’ dissertations in a typical African country where there is little or no training in research ethics in higher institutions of learning [9].

## Method

This is a retrospective descriptive study. Theses/dissertations on HIV research studies, which were available in university libraries in Cameroon, had been collected as part of an anterior study to establish an in-depth bibliography of studies on HIV/AIDS in Cameroon (Munung et al., unpublished). All theses/dissertations had been collected with the permission of the university authorities. Theses/dissertations describing research carried out in Cameroon but defended in a university out of Cameroon were not included in this study.

In the bibliographies, there were two hundred and sixty-one (261) student theses/dissertations on HIV/AIDS in Cameroon. These included theses/dissertations from 24 different universities (Munung et al., unpublished). The summary/abstract, “material and methods” section of each thesis/dissertation was carefully read and only theses that involved the use of humans as research participants or that reviewed personal medical records found in hospitals and clinics were included in the study.

For each thesis, we extracted information on the year the thesis was written, the type of academic degree for which it was written (Bachelors degree, Masters degree, an MD or a PhD). The theses were further grouped as Bachelor degree, Medical degree (MD) and a postgraduate degree (Maitrise, Masters, DEA, Doctorat and PhD). We checked each thesis/dissertation for a statement on research ethics approval and informed consent and, if available, the name of the REC or IRB from which research ethics approval was obtained. A similar method had been described in previous studies [2,19,21]. We also compared the reporting of these ethics parameters in the different degree types.

If ethics approval and informed consent were reported, we checked for the evidence (usually in the annex/appendix section of the thesis). For theses/dissertations that included the consent form, we assessed the content of the consent form using a pre-agreed checklist and also as previously described [20] with some modifications. Briefly, the checklist consisted of the following parameters: purpose of the study, the risks and discomforts associated with the study, potential benefits of enrolling in the study, provisions for confidentiality, if participation in the study was voluntary, right to discontinue participation in the study, the contact information of the researcher, the language of the consent form and provisions for the management of residual biological samples (where applicable). The types of benefits, risks, and contact details of the investigator as stated in the consent forms were also identified. No information that could identify the author of the thesis or dissertation was recorded.

## Results

**Figure 1:**
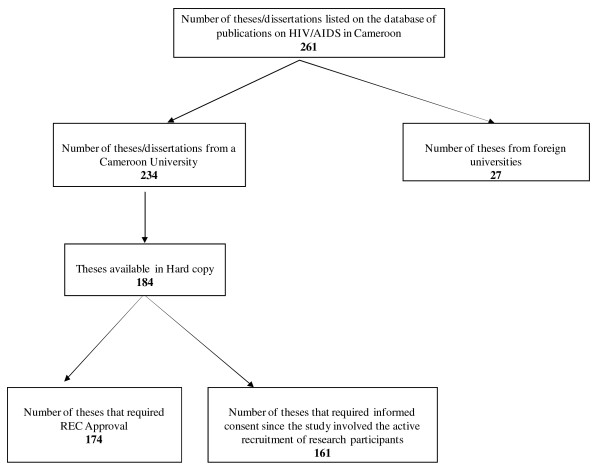
Flow chart indicating the number of theses/dissertations analyzed.

A total of 184 theses/dissertations on HIV/AIDS in Cameroon were included in this study. These theses covered 20 years of HIV research (from 1989 to 2010) from 4 universities in Cameroon (University of Buea, University of Ngaoundere, University of Yaoundé and the Catholic University of Central Africa). The areas of research varied and included clinical, social science, epidemiological and biological studies. Ten studies (5 in the years 2001–2005 and 5 in the years 2006–2010) did not require research ethics approval, as they did not involve the use of research participants. The analysis of the results on ethics approval therefore excludes these 10 studies. Of the 184 theses/dissertations, 23 studies did not require informed consent, as they either did not involve human research participants or involved only retrospective analysis of medical data/records. In total, 174 studies were included for analysis on research ethics approval, while 161 were included for analysis on informed consent (Figure 1). Per academic degree, the theses/dissertations analyzed included: 40 undergraduate dissertations, 62 MD theses and 27 postgraduate theses.

Overall, 17 (9.77%) of the theses/dissertations had a statement on research ethics approval of which 9 (52.94%) included a copy of the ethics approval certificate (Table 1). Informed consent was reported in 77 (47.83%) theses/dissertations and a copy of the consent form used in the study was included in 38 theses/dissertations (Table 2). Ethics approval was documented in 14.01% of the MD theses and in no undergraduate dissertations. Informed consent was reported in 64.58% (62 out of 96) MD theses and 11.11% (3 out of 27) postgraduate theses (Table 3).

**Table 1:** Research Ethics Approval and evidence of Ethics Approval in students’ theses/dissertations from 1986 to 2010

**Period**	**Number of theses/dissertations**	**Reported Research Ethics Approval (%)**	Attached proof of Ethics Approval letter(%)*
1986–1990	3	0 (0)	0 (0)
1991–1995	2	0 (0)	0 (0)
1996–2000	28	0 (0)	0 (0)
2001–2005	82	4 (4.88)	2 (2.44)
2006–2010	59	13 (22.03)	7 (11.86)
**Total**	**174**	**17 (9.77)**	**9 (52.94)**

*Percentage in this column indicates the proportion of theses that had a copy of the Ethics Approval used in the study to those that reported Research Ethics Approval.

**Table 2:** Informed Consent and evidence of Informed Consent in students’ theses/dissertations from 1986 to 2010

**Period**	**Number of theses/dissertations**	**Required Informed Consent**	**Reported Informed Consent (%)**	Attached Informed Consent form (%)*
1986–1990	3	3	0 (0)	0 (0)
1991–1995	2	2	0 (0)	0 (0)
1996–2000	28	25	11 (44)	2 (18.18)
2001–2005	82	79	41 (51.90)	18 (43.90)
2006–2010	59	52	25 (48.08)	18 (72)
**Total**	**174**	**161**	**77 (47.83)**	**38 (49.35)**

*Percentage in this column indicates the proportion of theses that had a copy of the consent form used in the study to those that reported informed consent.

**Table 3:** Ethics Approval and Informed Consent in students’ theses/dissertations according to degree type

**Academic degree**	**Required Ethics Approval**	**Documented Ethics Approval (%)**	**Required Informed Consent**	**Documented Informed Consent (%)**
Undergraduate	40	0 (0)	38	12 (31.58)
Medical Degree (MD)	107	15 (14.01)	96	62 (64.58)
Postgraduate	27	2 (7.41)	27	3 (11.11)
**Total**	**174**	**17 (9.77)**	**161**	**77 (47.83)**

**Figure 2:**
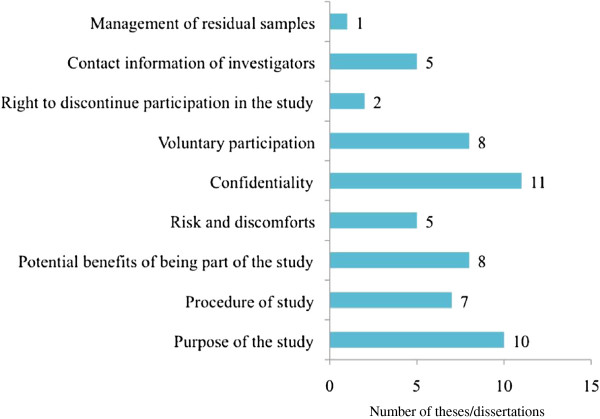
Content of Informed Consent forms as found in students’ theses/dissertations.

In all, confidentiality was stated in 11 consent forms and the right to discontinue participation in the study in 2 consent forms (Figure 2). The most cited benefit of participating in the study was the idea that the study would contribute to knowledge followed by free diagnosis of HIV and communication of results. Slight discomfort during sample collection from the patient was the most cited risk of participating in the study (Table 4).

**Table 4:** Analysis of the different benefits and risks found in Informed Consent forms

	**Nature of Benefits/Risks**	**Total**
**Nature of Benefits for participating in the study**	Contribution to general knowledge:	10
	Free diagnosis and communication of results:	8
	Improvement to patient care:	2
	Follow up of patient at no cost:	1
**Risks of participating in the study**	Slight discomfort during sample collection:	4
	Time used in participating in the study:	3
	Loss of blood:	1

Based on the data collected, students researching on HIV/AIDS in Cameroon and who had obtained ethics approval, did so from one the following RECs/IRBs: i) the Ethics Committee of the Faculty of Medicine and Biomedical Sciences, ii) the Ethics Review Board of the National Medical Council, iii) the National Ethics Committee and iv) the Ethics Review Committee of the Ministry of Public Health.

## Discussion

The results of this study indicate that students in Cameroon universities, involved in HIV-research, are not taking research ethics seriously into consideration when they design their research studies. And they could be said to be only kidding with research ethics even when they do take it into consideration, whereas the research itself they are doing is evidently no child’s play. This might be because they are either not sensitized on the importance of research ethics, are simply not aware of what research ethics is all about, or may be aware of the place and importance of research ethics but have deliberately decided to push it to the background for whatever reasons. It is important for stakeholders charged with university education to note that graduate students and students in medical schools constitute tomorrow’s scientists and therefore need to be properly trained in research methodologies. Such training should include HRE especially for those who are likely to become career scientists or to engage or participate in health related research. If this is done, it can realistically be expected that health research in Cameroon would conform to all the principles of good practice in the domain. Recent empirical studies have shown that researchers in Cameroon significantly failed to document research ethics approval in their publications [17]. Researchers working on HIV/AIDS have been cautioned on the need to respect the rights of people living with HIV/AIDS, especially as this group of persons has been classified as highly vulnerable. Furthermore, a ministerial regulation (Decree NÂ° 079/A/MPH/DS of October 22^nd^ 1987) governing health research in Cameroon demands that all health related research studies involving human research participants go through review by a REC. Students therefore need to be sensitized and encouraged to respect at least the basic research ethics procedures, namely, obtaining ethics approval/clearance from a REC before the commencement of a research study, obtaining genuine informed consent from research participants and ensuring confidentiality for people recruited into a study.

Despite the existence of a regulation that requires the submission of research protocols involving humans as research participants to a REC, and the creation of an ethics committee in Cameroon as far back as 1987, students in Cameroon still systematically failed, throughout the period under review, to obtain/document research ethics approval for their research studies. Only 9.77% of theses reviewed had a statement on ethics approval (Table 1). This is far below that recorded in a similar study (1985–2005) in another developing country (Sri-Lanka) where 34% of the students had a statement on research ethics approval in their theses [19]. The very first student thesis on HIV/AIDS in Cameroon was written in 1986 (Munung et al., unpublished). However, it was not until 2002 (16 years after) that research ethics approval was first documented in a student thesis/dissertation (Table 1). Since the first ethics committee in Cameroon was created in 1987, one would have expected that all research studies requiring ethics approval after 1987 would both obtain and document ethics approval. Documenting research ethics approval more than tripled in the last 5 years of our study, an indication that there is increased awareness amongst students and researchers in Cameroon on the need for having ethics approval from a REC before the start of a study. Nevertheless, it appears that relatively few students obtain ethics approval before the commencement of a research leading to the award of a an academic degree, considering that even the years 2006–2010 registered only 22.03% of theses with a statement about research ethics approval (Table 1). Though some (9.77%) students stated that they had obtained ethics approval for their studies, only 5.17% (9 out of 174) of the theses had provided evidence of such approval. In one of such few cases, the student stated having obtained ethics approval from one REC but attached evidence of ethics approval from another REC altogether. Based on the results of this study, it could be concluded that students in Cameroon universities are only kidding with research ethics, a situation that needs to be strongly addressed.

Respect for autonomy, one of the guiding ethical principles in research involving humans, requires that individuals be treated as autonomous agents, and that persons with diminished autonomy be granted appropriate protection (Belmont Report, 1979). This principle is applicable in research by ensuring that informed consent is appropriately obtained from all research participants. In this study, we realized that this principle appeared to be relatively better respected by research students when compared to, say, obtaining research ethics approval/clearance. Amongst the 161 studies that required obtaining informed consent, 47.83% had a statement on informed consent (Table 2). Although this corresponds to just half of the total number of theses/dissertations, the results show that students are more conscious of the requirement of obtaining informed consent, than that of seeking research ethics approval. A plausible reason for this awareness could be that a majority of the theses/dissertations reviewed come from students in medical schools (medical or other health science students) who already are acquainted with the importance of getting a patient’s consent for some procedures associated with medical care. However, documenting informed consent in theses/dissertations in Cameroon is relatively low when compared to other countries like Sweden [2] and Sri-Lanka [19]. This confirms the need to train Cameroon-based students in HRE as previously recommended by some authors [9,18].

Documenting ethics approval and informed consent varied tremendously across the various academic degree types. Most of the theses (14.01%) that had a statement on ethics approval were theses written for a medical degree, while no undergraduate theses documented ethics approval (Table 3). Similarly, there was a higher rate (64.58%) of obtaining informed consent amongst students studying for an MD than those studying for other degree types. Overall, postgraduate students (Masters, Maitrise, DEA and Doctorat) systematically failed to seek and document research ethics approval and informed consent. This might be because students in medical schools are more aware of such procedures, especially as protecting the rights of patients is an integral part of the Hippocratic Oath taken during convocation ceremonies. Maybe if a Hippocratic-like oath is taken by students in the biomedical sciences, the situation would improve [22].

Based on these results, including research ethics in the postgraduate curriculum is of utmost importance [20,23]. A previous study [17] had shown that, in addition to other ethics committees that are found in research institutions/organizations and hospitals around Cameroon, there exists, at least in name, a REC or IRB in almost all the state universities in the country. All the theses/dissertations that were included in this study were written in universities that had at least a nominal REC/IRB. It is therefore baffling as to why students still failed to seek ethics approval. The results suggest a need for education in research ethics even at the undergraduate level. This is justified on the basis that most health and allied science undergraduates in Africa do not proceed to the postgraduate level but might get involved in research during clinical practice or in research carried out by others [23].

The Ethics Committee of the Faculty of Medicine and Biomedical Sciences, the Ethics Review Board of the National Medical Council, the National Ethics Committee and the Ethics Review Committee of the Ministry of Public Health were the 4 ethics committees most cited in the theses/dissertations reviewed. This would seem to imply that students do not usually approach the RECs/IRBs in their own universities or faculties for ethics review, or else the RECs/IRBs in these universities might not be functioning properly. Whatever the case, should RECs/IRBs in the universities where students are based not be functioning, it is advisable that students submit their research proposals to the other functioning RECs/IRBs. Students and theses supervisors need to be sensitized and encouraged on the importance of obtaining ethics approval before the commencement of any research study that involves human research participants and/or access to personal medical data. Such sensitization can be achieved through organization of training workshops in HRE for researchers/investigators in Cameroon and the teaching of research ethics in universities.

In this study, only 49.35% of the theses reviewed had a sample of the informed consent form that was used for the recruitment of research participants (Table 2) and the first thesis that included the informed consent form was written in 1997. In analyzing the content of the informed consent forms we realized that most were very similar in word and content. A typical consent form as found in most theses/dissertations reads as follows:

"I…………. have understood the objectives of this study titled [title] and have accepted freely to be a participant in the study."

Further analysis of the consent forms revealed that explaining the purpose of the study and that confidentiality would be protected were a common aspect in the consent forms, followed by explaining the potential benefits of being part of the study. The right to discontinue participation in the study and the management of remaining study samples were scarcely mentioned in the informed consent forms (Figure 2). This could be due to fear on the part of the students that giving a potential research participant the idea that he/she could withdraw from the study at any time might act as a self-fulfilling prophecy, thereby making it difficult, if not impossible, to reach the target sample size. This is similar to the results obtained in a study on informed consent forms used in genetic research in Oman, in which withdrawal of samples by participants was rarely mentioned in informed consent forms [24]. Contribution to ‘generalisable knowledge’ was the most cited benefit for participating in the study, while ‘slight discomfort’ during sample collection was the most cited risk of participating in the study (Table 4). Though not an objective of this study, we realized that some theses had a list of the names of research participants. While this was probably done in the interest of transparency or acknowledgment, it is quite surprising that the implications for confidentiality, especially in studies of such highly stigmatizing conditions as HIV/AIDS, did not seem to be realized, let alone considered.

## Conclusions

Health related research in Africa still faces a lot of ethical hurdles which can be successfully crossed only if capacity building in the continent involves harmonized training of young researchers, (especially university students) in HRE. Such training should be accompanied by the training of members of RECs. This study demonstrates that students do not seem to consider research ethics, or at least its reporting and documentation, as important, since they significantly failed to report ethics approval and informed consent in their theses, although a slight progressive improvement is observable in the last 5 years. The main reasons for this situation could be that i) there is no systematic training in research ethics in Cameroon universities and ii) supervisors/senior researchers do not appreciate or are not aware of the importance of research ethics and, consequently, fail to mentor their students or trainees appropriately about it or they simply push it to the background. It is therefore essential that training in HRE be incorporated in the curriculum of universities in Cameroon in order to bring up the next generation of scientists equipped with a thorough knowledge and practice of HRE. Also, guidelines for writing student theses should include at least a statement requiring students to show proof of ethics approval in their theses, if the research involves human participants. This, we believe, would be one way of fighting the occurrence of research scandals, many of which probably result from negligence and ignorance rather than from deliberate intention. Equally recommendable is the introduction of standardized continuous education and refresher courses in research ethics for both junior and senior researchers in Cameroon. If such measures are not put in place, the country surely runs the risk of documenting more research scandals in the nearest future.

## Competing interests

The authors declare that they have no competing interests.

## Authors’ contributions

NSM conceived the study, participated in the design of the study, collection and analysis of data and drafting of the manuscript. GBT participated in the design of the study, analysis of the data, coordination of the study and drafting of the manuscript. CPC was involved in the design of the study, collection of data, analysis of data and in drafting the manuscript. LV was involved in study design, analysis of data and drafting of the manuscript. OOMOB participated in the design of the study and coordination, data collection and analysis, and the writing of the manuscript. All authors read and approved the final manuscript.

## Pre-publication history

The pre-publication history for this paper can be accessed here:

http://www.biomedcentral.com/1472-6939/13/12/prepub
